# Explainable AI approach with original vegetation data classifies spatio-temporal nitrogen in flows from ungauged catchments to the Great Barrier Reef

**DOI:** 10.1038/s41598-023-45259-0

**Published:** 2023-10-24

**Authors:** Cherie M. O’Sullivan, Ravinesh C. Deo, Afshin Ghahramani

**Affiliations:** 1 University of Southern Queensland, Toowoomba, QLD 4350 Australia; 2 School of Mathematics, Physics and Computing, University of Southern Queensland, Springfield, QLD 4300 Australia; 3 Center for Applied Climate Sciences, University of Southern Queensland, Toowoomba, QLD 4350 Australia; 4 Present Address: Department of Environment and Science, Queensland Government, Rockhampton, QLD 4700 Australia

**Keywords:** Hydrology, Ecological modelling

## Abstract

Transfer of processed data and parameters to ungauged catchments from the most similar gauged counterpart is a common technique in water quality modelling. But catchment similarities for Dissolved Inorganic Nitrogen (DIN) are ill posed, which affects the predictive capability of models reliant on such methods for simulating DIN. Spatial data proxies to classify catchments for most similar DIN responses are a demonstrated solution, yet their applicability to ungauged catchments is unexplored. We adopted a neural network pattern recognition model (ANN-PR) and explainable artificial intelligence approach (SHAP-XAI) to match all ungauged catchments that flow to the Great Barrier Reef to gauged ones based on proxy spatial data. Catchment match suitability was verified using a neural network water quality (ANN-WQ) simulator trained on gauged catchment datasets, tested by simulating DIN for matched catchments in unsupervised learning scenarios. We show that discriminating training data to DIN regime benefits ANN-WQ simulation performance in unsupervised scenarios ( *p*< 0.05). This phenomenon demonstrates that proxy spatial data is a useful tool to classify catchments with similar DIN regimes. Catchments lacking similarity with gauged ones are identified as priority monitoring areas to gain observed data for all DIN regimes in catchments that flow to the Great Barrier Reef, Australia.

## Introduction

Communicating catchment influences towards the ecology of the receiving environment is enhanced by water quality simulation tools. Customising water quality simulation models to the catchment they represent is essential for limiting uncertainty in results and maintaining trust in land use decisions they aim to inform^1,2^. Model design and development, referred in here as customisation, is achieved by using observed water quality data from gauging stations for design and verification of models^3^. However, many water catchments globally are ungauged, and a lesser proportion of those have corresponding water quality data to inform model customisation. Techniques to overcome such data voids in ungauged areas are necessary^4,5^. Methods to simulate flows in ungauged areas are well researched^6,7^, however, refinement of methods that simulate nutrients in ungauged areas remained unresolved. This knowledge gap in water quality modelling needs addressing to best inform anthropogenic nitrogen management, and to demonstrate progress to the 2030 UN Nations Sustainable Development Goals commitment to reduce land-based nutrients that enter the oceans^8^. This has relevance for the Great Barrier Reef World Heritage Area where over ~ 20% of the terrestrial drainage area is ungauged, and nutrient balances are critical for the reef’s health^9,10^. Logical explainability in nutrient models for ungauged areas can support communications and enable more responsive water quality improvement investments^11,12^.

For both data-driven and process-based models that simulate water quality, observed water quality and quantity data, as well as a comprehensive understanding of catchment characteristics are required^13^. Data driven water quality models are useful to forecast water quality output, but water flows and water quality must be known a priori to develop covariates^14,15^. In contrast, process-based models use physical and empirical principles and can be established for catchments lacking observed water quality data. In ungauged areas, data is donated to ungauged catchments from the most similar gauged ones^16,17^. Alongside these traditional water quality modelling approaches, deep learning, particularly in the revised forms of Artificial Neural Networks, has been relatively successful in simulating water quality, including nitrogen, without the need for prior established principles^18,19^. As a subsector of Deep Learning, Artificial Neural Networks have demonstrated the ability to recognise patterns in input datasets, classify them, and establish algorithms to match target data. The merits of ANN are demonstrated to forecast and extend non-linear water quality data within respective catchment datasets^20^, but their application to inform scenario simulation, and hence land management decisions, which is the benefit of process-based models, is lacking^21^.

To exploit the benefits and overcome the drawbacks of each data driven vs process based model approach, the coupling of machine learning models such as ANN with process-based approaches can be performed to provide benefits of transfer learning^22,23^. However, machine learning models that incorporate process considerations for water quality modelling are disproportionally underrepresented in many research articles^22,23^. Additionally, where applied to ungauged areas, low landscape heterogeneity between drivers for the constituent being simulated is necessary^21,24^. While variations in patterns of nutrients are observed across gauged catchments that drain to the Great Barrier Reef^15,25,26^, methods for classifying those catchments to the most similar ungauged catchments that drain to the Great Barrier Reef, based on similarity of nitrogen drivers, are unexplored.

In terrestrial landscapes, Dissolved Inorganic Nitrogen molecules are influenced by decomposers, vegetation uptake, nitrogen fixing bacteria etc., which change depending on a unique combination of physical and biological influences at each location^27,28^. The fluxing nature of these biotic processes mean catchment similarities for drivers of DIN differ from the abiotic drivers of flow have therefore been complicated to quantify^22,29^. Variability in drivers of DIN affect the consistency of water quality modelling of ungauged areas^25^. This disparity between biotic and abiotic influence on nitrogen drivers means classical classification approaches that only use physical similarities miss the influence of all biologically influenced differences that may exist between catchments. Spatio-temporal variability in nutrient drivers can be represented in catchment models by the natural physical drivers of geology, aspect, topography, climate etc., as well as land use to represent anthropogenic impacts, including standard fertiliser application rates which affect DIN^25^. Our earlier studies found Original Vegetation is a proxy dataset for the residual biotic responses to these, and any other unaccounted-for drivers that can parsimoniously classify catchments for DIN, and identify the classification drivers using explainable artificial intelligence, (XAI)^30,31^.

Explainable artificial intelligence, (XAI) has outstanding capabilities to highlight the influential variables in machine learning algorithms, however, performance criteria for the corresponding ANN models are likely to vary unpredictably with changes to model architecture and scenarios^32^. Established process-based models instead can be customised to respective catchments using regionalised parameter data, enabling trials of different land management scenarios^18^. This technique has been effective for water quality constituents driven by abiotic processes, which result in consistent performance^6^, and so is pragmatic for the purpose of informing land management decisions. Despite the acceptable track record for process-based models, the suitability of parameter transfer for constituents with biotic process drivers is still lacking, and studies regarding the spatio-temporal scales are necessary^33–35^. We found earlier that original vegetation can be a proxy for matching gauged catchments with dynamic DIN patterns^26^. However, no approach has yet been developed that matches ungauged to gauged catchments for DIN similarities, which would be beneficial for models that transfer data across catchments with similar processes.

This study extends our previous XAI-SHAP^30,36^ approaches to match the currently ungauged to gauged catchments that flow to the Great Barrier Reef using mapped spatial data as a proxy for DIN. Mapped spatial data is useful because it provides data for all areas of the Great Barrier Reef catchments where water quality data is lacking^26^. In this study we verify the classification results by building and applying an ANN-WQ simulator to compare changes in simulation performance criteria for a case study catchment, under various dataset arrangement scenarios. Our earlier studies found dominant original vegetation data features may provide guidance to the part of the hydrograph that is relevant to consider for matched catchments and that it is a useful proxy to group gauged catchments that flow to the Great Barrier Reef to three DIN response categories^30^. In this study we evaluate whether our previous method is extendable to all ungauged catchments that flow to the Great Barrier Reef, and undertake a case study to verify its suitability as a proxy for DIN classification. Our verification case study aims to confirm catchments classified together based on original landscape variables also have transferrable water quality responses that can be exploited to simulate DIN.

The hypotheses investigated here are: (1) Original vegetation spatial features found to be a proxy for DIN discharge from gauged catchments in our previous studies^26,30^ can be used to match gauged catchments to ungauged catchments that also flow to the Great Barrier Reef. (2) An ANN-WQ simulator trained using predictor variables of original vegetation, coupled with flow data characterised to match with DIN targets will achieve superior performance compared to an ANN-WQ simulator trained to simulate DIN using non-categorised flow data only.

(3) The trained ANN-WQ simulator can simulate DIN in an unsupervised scenario for a pseudo-ungauged case study catchment matched based on the spatial proxy data and achieve satisfactory performance criteria to verify the suitability of the catchment match approach. For this study, pseudo-ungauged means a gauged catchment, with the same data collection method as the other gauged catchments, but intentionally omitted from previous research that informed this study. DIN data for the pseudo ungauged catchment is used here for hypothesis validation purposes only.

The present study therefore aims to create an XAI approach for considering original vegetation data classification as the proxy for spatio-temporal nitrogen patterns in ungauged catchment flows for the specific case of the Great Barrier Reef in Australia.

## Results

### ANN-PR matches

**Figure 1:**
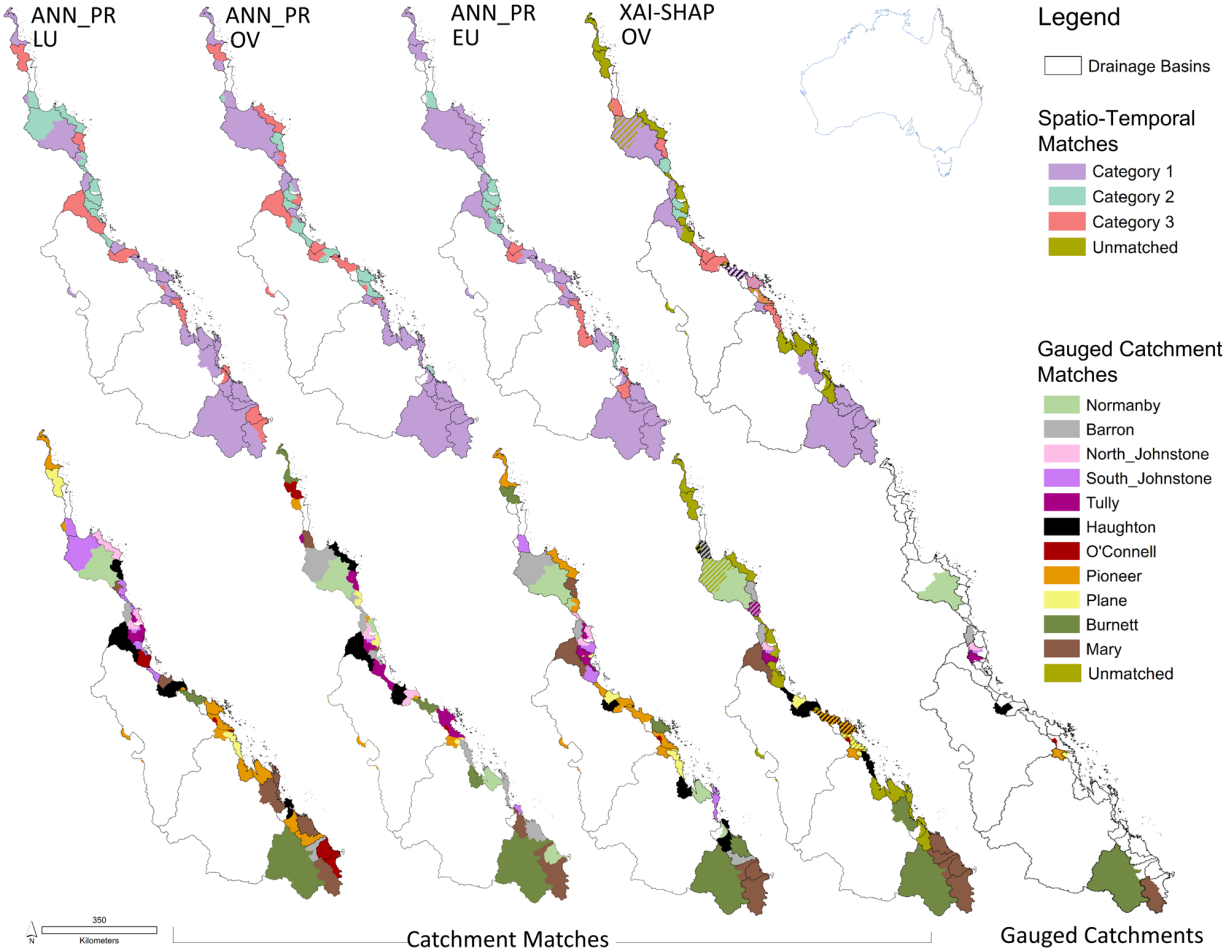
Catchment matches using ANN-PR and XAI-SHAP approach to identify ungauged catchment similarities to gauged catchments using spatial dataset (OV original vegetation, EU ecounit, LU land use). Top row shows the spatio-temporal category of the matched gauged catchment based on the gauged catchment allocation derived from our previous works^30^, bottom row shows the matched catchment. Colours represent the gauged catchment as listed in the legend. Results show high variation between each dataset. Maps created by author using ArcMap 10.8.1, gauged catchments^10^ supplied, Drainage Basins^37^ licenced under a Creative Commons—Attribution 3.0 Australia licence (CC BY 3.0 AU). © State of Queensland (Department of Environment and Science) 2023.

Apart from the Mary Catchment, the results show that ungauged portions of gauged catchments do not necessarily classify together, and catchments do not necessarily classify with their nearest neighbours (Fig. 1). Catchment matches varied for each spatial dataset evaluated, and translation of those results to classify catchments based on corresponding DIN response Categories also varied (Table 1). While Category 2 matched catchments generally clustered together spatially, Category 3 matched catchments contrasted with distributions only north of Plane for the Original Vegetation (OV) dataset compared to further south where Land use (LU) variability was included independently or embedded within the Ecounit (EU) data. This indicates that the catchments in the different datasets show different spatial characteristics. For example, the catchments that matched Category 2 tended to be clustered together, while the catchments that matched Category 3 showed more variability when the LU dataset, which represents anthropogenic, in contrast to natural biotic response to environmental influences, was included.

**Table 1:**
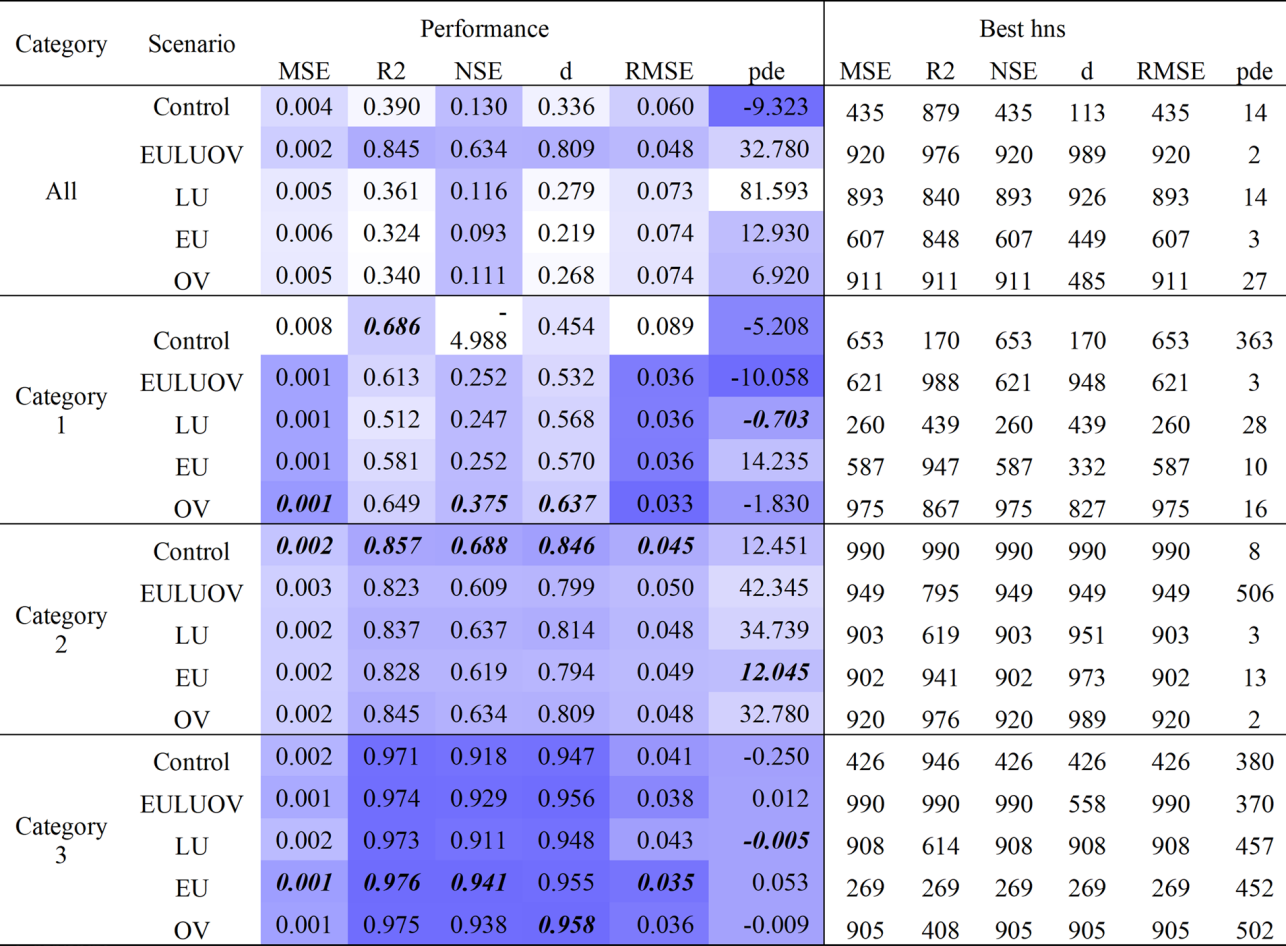
Performance evaluation of ANN-WQ simulator for the Gauged Catchment scenarios.

Shading intensity represents performance over all scenarios tested for each criteria. Scores styled **bold** are the best performing metric for each category, scores styled *italic* fail to meet minimum satisfactory performance criteria. Training datasets discriminated by category influence DIN simulation results.

### Variable feature independence

In this study, matching variable deviations using the XAI-SHAP approach method^30^ revealed that every catchment had a unique combination and weighting of deviated features. The same combinations of top XAI-SHAP 10% floristic structure variables did however match the most similar gauged catchment and group them to Categories based on the combination of deviated variables. It also revealed catchments that did not share the same combinations of deviated variables. Grouping the deviated variables by landform and vegetation descriptors in the Original Vegetation dataset allowed for 20 of the 37 pseudo/ungauged catchments to be matched to individual gauged ones, while 9 catchments were not matched to another ungauged or gauged catchment or spatio-temporal category. Of those, unable to match to gauged catchments XAI-SHAP results facilitated four closely matched groups to be identified.

**Figure 2:**
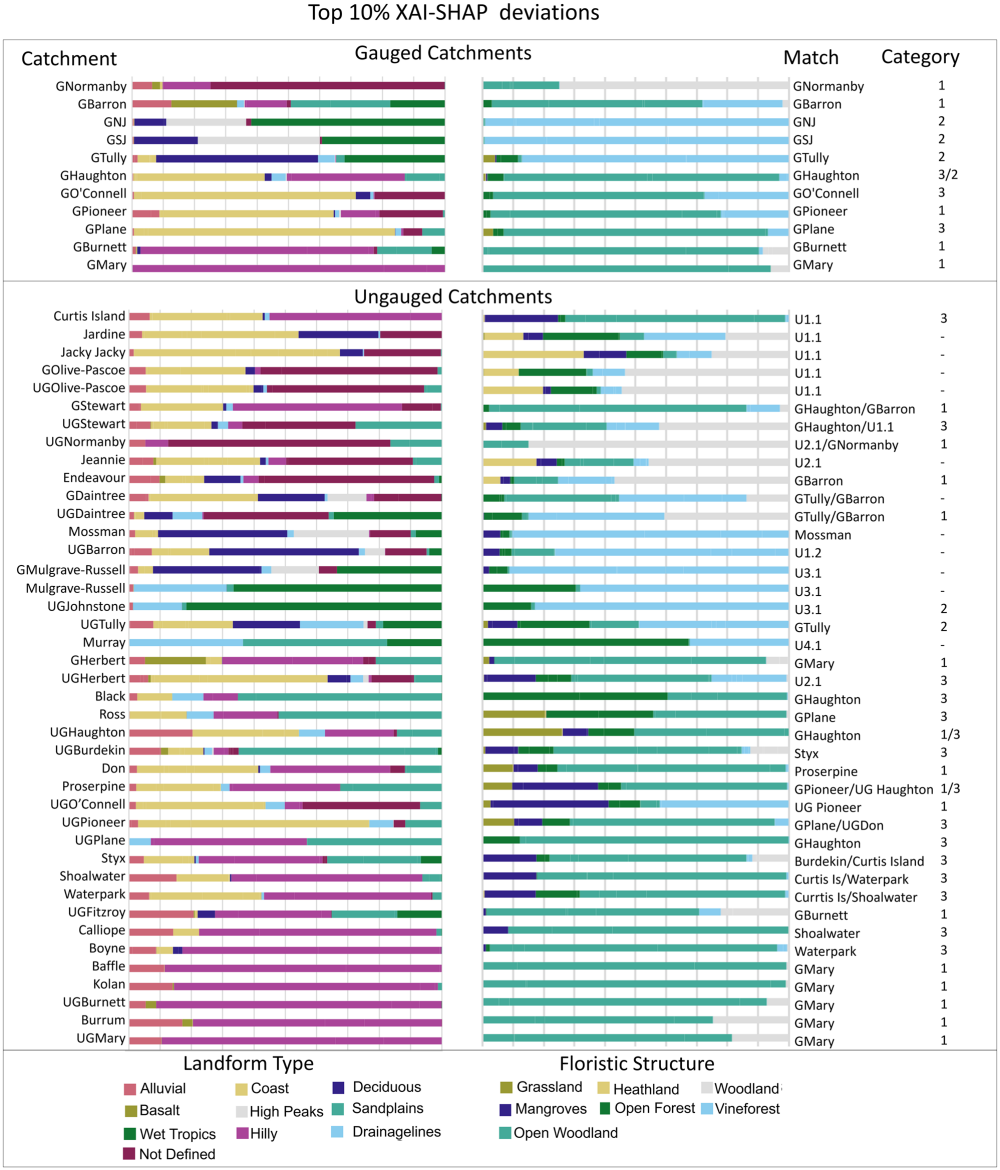
Top 10% XAI-SHAP deviations for landform and flora sub-descriptors of the Original Vegetation Datasets. Catchment = subject catchment, Match = Catchment the subject catchment is deemed a closest match with, Category = spatio-temporal category of the gauged catchment as established by previous research^30^. Gauged catchments are shown in the top plates, the ungauged catchments are shown in the bottom 2 plates and arranged chronologically from north to south. Visualisation of this data shows that some catchments have combinations of similar feature deviations to gauged catchments, and others are unique.

Variable combinations only occurring in ungauged catchments and not in the gauged ones include: hilly alluvial with basalt, health land with sandplains and coast, or mangrove landform structures, as well as additional combinations of vineforest with woodland drainage, or open forests combined with grassland and open woodlands (Fig. 2).

### ANN-WQ simulator performance

The most notable observation was that the combination of catchments included in the training datasets influenced the unsupervised performance of the ANN-WQ simulator (Table 1). When the ANN-WQ simulator was trained using data for individual catchments, simulations were only able to be generated in the unsupervised environment for the Wet Tropic catchments of Tully, and North and South Johnstone. Flatline simulations were observed in the unsupervised simulator environment for all other catchments, despite their adequate training performance (Supplementary Material Fig. SF1).

In contrast, training using data grouped from multiple catchments generated non flatline results for all scenarios. Satisfactory to very good performance for all metrics were achieved for all spatial dataset combinations grouped and discriminated in spatio-temporal Category 2 and 3. Except for their unsatisfactory Nash Sutcliffe Efficiency (NSE) performance, datasets grouped and discriminated to spatio-temporal Category 1 also achieved satisfactory to very good performance (Table 1). Meanwhile, training datasets that grouped all gauged catchments together only met satisfactory performance criteria for the scenario that included all spatial data variables (i.e., Ecounit, Land use and Original Vegetation (EULUOV)) (Table 1).

Performance criteria for the control (i.e., flow only) scenario also varied where the dataset was first discriminated to spatio-temporal categories (Table 1). Simulation results for the control scenario trained on non-discriminated datasets failed performance criteria (NSE = 0.130), while the opposite was the case for training datasets discriminated by spatio-temporal category (NSE = 0.846 and 0.947 for Category 2 and 3 respectively). The NSE for Category 1 catchment Control worsened after discrimination, however, the R^2^ value improved to 0.686 compared to 0.39 for the non-discriminated counterpart. Benefits of including spatial data in datasets were reduced after pre-discriminating to spatio-temporal regime (Table 1). Benefit losses include a lack of independence from the control scenario, as measured by Kruskal Wallis test for independence (*p* = 0.483–0.981), where spatial data was omitted (Supplementary Material Table ST1).

Grouping datasets by respective catchment categories, identified in our previous spatio-temporal study^30^, prior to loading to the DIN simulator resulted in improved performance criteria (R^2^ = 0.984 for Category 3, RMSE = 0.02382 for Category 1). Interestingly, for Category 2 flow datasets the control scenarios, which did not contain spatial variables achieved superior performance for MSE, R^2^, NSE and Wilmott’s d compared to the other Category 2 scenarios that did include information on spatial variables. In contrast, the Original Vegetation scenario discriminated to Category 3 records had the smallest pde score meaning that the inclusion of Original Vegetation variables improved the ability of the DIN simulator under extremes in the data for Category 3 catchments (Table 1).

### Classifying ungauged to gauged catchments: variable independence vs ANN-PR

While the ANN-PR approach matched all ungauged catchments to a gauged counterpart, the XAI-SHAP variable independence approach using relative variable distributions was unable to match 17 catchments. Catchment matches using OV dataset for XAI-SHAP landform and floristic structure, most closely aligned to the ANN-PR catchment matches using the EU dataset. Matching using only the top 10% of deviated features using XAI-SHAP variable independence approach changed the catchment matches compared to the EU dataset using ANN-PR, where all variables are considered, but retained matches generally within the same category (Fig. 1).

### Verification of catchment classification for DIN similarities

**Figure 3:**
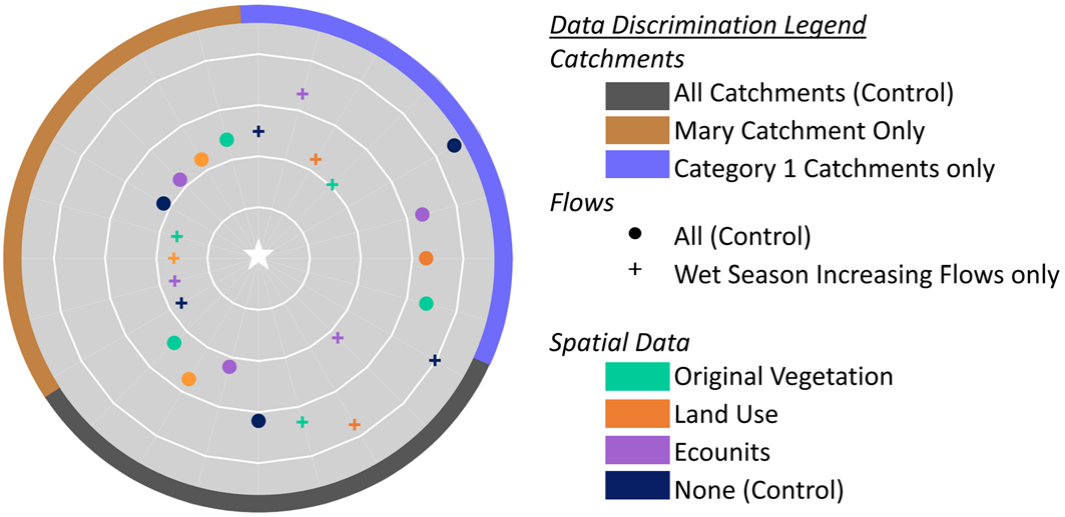
Additive Performance Criteria for pseudo-ungauged catchment DIN simulations. Dimensionless graph shows the additive scores for the best MSE, R^2^, NSE, d, RMSE for each data discrimination scenario evaluated in the case study. Zero is the centrally located white star and represents the target/observed data. Discrimination of data to spatio-temporal regime (i.e., shown as crosses) and to the matched catchment improves results the most. Inclusion of Ecounit data improves simulation performance where training datasets are not discriminated to classified catchment or category.

Both XAI-SHAP Variable Independence and ANN-PR techniques for catchment classification matched pseudo-ungauged Herbert to the Gauged Mary (Figs. 1 and 2) and identified it as a Category 1 catchment. Only Mary catchment training data scenarios achieved a satisfactory performance metric i.e. NSE > 0.5 (Supplementary Material Table ST2). The greatest performance criteria overall collectively clustered towards datasets discriminated to Mary and Category 1 flows only (Fig. 3 and Supplementary Material Table ST2).

Datasets first discriminated by the classified catchment resulted in the best overall performance, with further discrimination to flow regime improving results. Training datasets discriminated by spatio-temporal flows, also performed better where they were also discriminated to the flow regime. This is consistent with findings during the development of the ANN-WQ simulator where a significant difference was noted for training datasets discriminated by the category flow regime. Where catchment specific or catchment category classification was not included, performance improved the most for training datasets that included Ecounit spatial data compared to the control which did not include spatial data. The worst performing scenario was the control grouped to Category 1 catchments only, followed by the control for all gauged catchments that included no spatial data, but was discriminated by flow regime. In comparison to control scenarios, differences in the performance criteria for scenarios that include spatial data diminished for catchments trained only to Mary gauged data. This suggests the benefit of adding spatial data reduced as the flow regime was refined to the catchment with the closest similarity to Herbert.

Training data discriminated to the individually matched catchment, Mary, and discriminated to wet season flows achieved the best performing DIN simulations (R^2^ = 0.80, NSE = 0.62, d = 0.85 respectively). Visualisation of simulated vs true data demonstrates that these scenarios’ pre-discriminated spatio-temporal flows result in simulations that include all the peaks in the observed dataset. On the other hand, training data discriminated to include all catchments in the corresponding Category 1, but using all flow and season records, with no spatial data failed to simulate half the peaks (Supplementary Material Fig. SF2). While simulated peaks were under estimated in all cases, a review of the raw data identified that the maximum nitrogen concentration in the dataset for Herbert Catchment was 1.8105 mg/L, which is the highest historical record, plus two additional peaks ranging between 1.320 mg/L and 1.694 mg/L. Maximum concentration for Mary was a smaller with a once off observed peak of 1.243 mg/L during unusual weather conditions of end 2012 start 2013^38,39^ with remaining peaks in the dataset not exceeding 0.605 mg/L.

## Discussion

### Overview

Our research uniquely evaluates the classification potential for all ungauged catchments flowing to the Great Barrier Reef, based on proxy data for spatio-temporal drivers of Dissolved Inorganic Nitrogen (DIN). We adopt an explainable AI approach referred to as XAI-SHAP to provide a deeper understanding of the modelled classification results. In accordance with earlier research works, our satisfactory performance metrics show classification of the pseudo-ungauged area to the most similar gauged ones is validated and works well where data for proxy drivers of DIN are included because they facilitate grouping of catchments by the DIN regime. Evaluation of DIN simulation performances using transfer learning in an Artificial Neural network environment allowed us to demonstrate the variability in DIN patterns depending on the spatio-temporal regime of the ungauged catchments, as exposed by original vegetation data Additionally, the XAI-SHAP method allowed for ungauged catchments with insufficient similarity to the gauged ones to be identified, regardless of being classified by brute force using ANN-PR techniques.

### Dataset complexity and consistency

Development and verification of the ANN-WQ simulator to establish DIN response similarities in datasets between pseudo-ungauged catchments with the gauged ones found dataset complexity and representative flow patterns were influential. This highlighted caution in direct application without prior understanding of the DIN to flow dynamics of the catchment. Flatline simulations that resulted in the unsupervised scenario are a known symptom of inadequate complexity in the dataset^40^. Likely explanations include hidden neuron complexity was low in the development trials and relationships between flow, spatial data and DIN response was not adequately formed to facilitate simulations in the unsupervised scenario. The contrasting ability of Wet Tropics catchment datasets to overcome possible lack of dataset complexity in the training dataset is explained by the different DIN and flow dynamics in wet tropics catchments compared to the others^27^. We previously demonstrated that DIN remains elevated in retreating flows for Wet Tropics catchments only^26^. One explanation for the contrast with Wet Tropics catchments could be a more consistent relationship between flows and DIN releases throughout the hydrograph which the ANN-WQ simulator was trained to simulate for^41,42^. This phenomenon demonstrates that consistency of DIN to flow relationships influence the performance of defined algorithm based models developed to use transferred data.

### Training dataset influence

For catchments with inconsistent DIN to flow relationships, our results found training data arrangements that group catchments using prior knowledge of spatio-temporal similarities, i.e. either by prior discrimination (discriminated to Category 1, Category 2 and Category 3 as informed by Original Vegetation deviation using XAI-SHAP), or within the model training datasets (non-discriminated but including all EULUOV spatial variables which are identified as proxy drivers for DIN and used to inform XAI-SHAP) improved the performance. This approach to remove heteroskedasticity where seasonal differences for Nitrate are considered has already been shown to benefit model development^42,43^. The significant differences in performance criteria of DIN simulations (*p* = 0.003–0.045) depending on the data discrimination for catchment categories suggests that DIN dynamics differ between those categories. This finding of significant variation in nitrogen regimes through the Great Barrier Reef catchments, as demonstrated by the ANN-WQ simulator training dataset predictive performance, regardless of anthropogenic influence is consistent with proceeding research^34,44,45^. Our research shows variability in DIN regimes is an influential consideration for data transfer purposes in water quality models. The improved performance criteria where information on proxy drivers of DIN was considered supports our application of original vegetation spatial datasets used in this study to discriminate differences in DIN regimes for each catchment^30^.

Spatio-temporal category differences for DIN simulation performance may be explained via the wide body of literature that demonstrate nitrogen is either flow or production limited^44,46,47^, and also influenced by connectivity to stream network^48^. The superior performance of the control scenario for Category 2, compared to Category 2 scenarios that included spatial data, indicate this category is flow limited and abundant in DIN. It is demonstrated that soils higher in total organic carbon, consistent with rainforest soils, have higher supplies of nitrogen created by the residual soil biology^30,49,50^. The abundance of nitrogen generation in the soils, coupled with abundant flows, in the Wet Tropics catchments can result in consistent nitrogen to flow patterns and is a logical explanation for the ability of the ANN-WQ simulator to generate results in the development trials, where catchments from other categories flatlined. This supports our previous suggestions^30^ that the timing of data collection is important in Category 1 and 3 catchments, while Category 2 catchments could classify regardless of the season or flow phase. While our research is not designed to interrogate reasons for drivers of DIN in each category per-se, this is one of many possible explanations for how categorising datasets by vegetation removes noise associated with different combinations of biotic responses in each location^34,41,51^. The findings, therefore, support the second hypothesis that prior grouping of catchments by categories of Original Vegetation, as a proxy for the DIN to flow regime, is a necessary first step for identifying catchments that share similar DIN patterns.

Training dataset discrimination and variable combinations separately influenced the performance of the ANN-WQ simulations of DIN for the pseudo ungauged catchment. The ANN-WQ simulator achieved the best DIN simulation performance metrics for the pseudo-ungauged catchment when trained on data only from the classified catchment and therefore highlights that data transfer with the classified catchment achieved the best results. Concurrently, discriminating the dataset by the respective flow regime of wet season increasing flows had a greater influence on simulation performance than inclusion of spatial data variables. In contrast, training datasets using data from multiple catchments from the same flow regime, i.e. Category 1, achieved equivalent performance only where the training data was first discriminated to increasing above average flow regime, hence removing hetroskedacity of DIN in the retreating and below average flows. Both these findings are consistent with the ANN-WQ development phase and demonstrate that prior discrimination of the training dataset to flow regime reduces heteroscedasticity in DIN patterns to flow^41,52^. Once heteroskedasticity in the training dataset was removed, the influence of spatial variables as drivers to the DIN patterns became less relevant. Separately, the research also found that training the ANN-WQ simulator using data from all catchments improved where all EULUOV variables were included. This could be attributed to the ANN-WQ simulator discriminating datasets within the algorithms, as opposed to prior discrimination provided by classification, and further demonstrates the benefit of the spatial datasets to expose the drivers of the DIN patterns.

### ANN-PR vs XAI-SHAP classification

Catchments matched using ANN-PR were not always the same as the catchments recommended to be matched by the XAI-SHAP deviation approach for variable independence. One reason could be that only 10% of the most influential variables were considered in the XAI-SHAP approach, in contrast the less deviated variables contributed to the ANN-PR matches. Catchment classification informed by the match options in both ANN-PR and XAI-SHAP approaches provide foundational guidance to rationalise catchments to evaluate in future data transfer investigations or models for DIN simulations to the Great Barrier Reef^53^. Varied performance for each scenario trialled in the ANN-WQ simulator development phase demonstrated that training data from catchments with the most similar proxy drivers of DIN dynamics, was more suitable for data transfer compared to training data from all catchments lumped together^54^. This demonstrates that rationalising training data to the most similar responding catchments reduces heteroskedasticity in the training dataset and benefits DIN simulation accuracy for the classified catchment. XAI-SHAP provided insight to identify catchments grouped by known DIN to flow proxy drivers. While classification using nearest neighbour catchments has historically been supported for their influence towards flow similarities^15,55^, our finding demonstrates that catchments with the most similar drivers of DIN, in addition to flow, are not necessarily located as the neighbouring catchment, and are influential towards DIN simulation performance.

### Practical application

This study established Original Vegetation as a suitable proxy for DIN dynamics for the benefit of water quality modelling. Therefore, the 20 ungauged catchments that matched to gauged ones, based off Original Vegetation similarity have justification to receive data from the corresponding gauged catchment. The remaining 21 ungauged catchments had combinations of original vegetation unique from the gauged catchments and therefore did not support the hypothesis that they share similar DIN drivers with gauged catchments. Consequently, this study found that only 20 of the 41 ungauged catchments were suitable to consider for data transfer with existing gauged catchments for satisfactory water quality modelling purposes.

Of the ungauged catchments that failed to match to gauged ones, 4 groups shared unique combinations of deviated spatial variables. Deviated original vegetation floristic structure and landform descriptors shown by the XAI-SHAP deviations, were grassland, heathland and mangrove. These are all coastal ecosystems and differ from vineforest, open woodlands and forest shown to be proxy indicators of DIN dynamics for gauged catchments^30^ While data transfer from existing gauged catchments to the four coastal catchments is not supported by our study, our method can instead be used to inform where new water quality monitoring and gauging sites could have the greatest value to represent all DIN regimes^12,47,56–58^. New monitoring and gauging sites are recommended in each of the four coastal catchment groups to collect data representative of all DIN regimes, which could facilitate data transfer for modelled DIN predictions across all ungauged Great Barrier Reef catchments.

It is well known that performance of neural networks deteriorates when the unsupervised scenario includes extremes outside the range of the training dataset^59^ and in our evaluation, models trained using Mary data were never exposed to high concentration peaks observed in the Herbert catchment. Limitation for simulating extremes not included in the training data could be addressed with differing model techniques, the ANN-WQ simulator was intended only as a coarse method to verify whether similarity in DIN drivers exists between catchments matched using the ANN-PR method, and this was demonstrated.

For the case study, the matched catchment was a Category 1 catchment. Collectively, Category 1 catchments showed the poorest performance in the ANN-WQ development phase. The fact the case study trial achieved satisfactory performance criteria for the poorest performing category in the development phase, it is expected that better results can be achieved for Category 2 and Category 3 catchments which have less heteroskedastic DIN to flow relationships. Further studies to refine the ANN-WQ simulator performance, along with a full comparison of all training dataset options, i.e. discrimination of data to Category 2 and 3 flows to evaluate difference in performance and facilitate year round classification is recommended. Regardless of these limitations, we encourage results from this study to be applied in established models that will benefit from data transfer from the most similar catchments for purpose of DIN modelling, and intentionally developed for superior performance^43^.

## Conclusions

This study matched all ungauged catchments that drain to the Great Barrier Reef to the gauged ones using ANN-PR coupled with Land use and Original Vegetation datasets. While ANN-PR enabled matching using proxy datasets for drivers of DIN, XAI-SHAP method explained similarities between catchments based on feature deviations as well as concurrently allowing grouping of catchments to known spatio-temporal categories. Prior knowledge of spatio-temporal DIN response categories within training datasets improved performance of the ANN-WQ simulator developed to verify catchment matches.

While all catchments matched to a gauged one using ANN-PR, consistent with hypothesis 1, the additional interrogation by XAI-SHAP deviations found 17 catchments did not share deviated feature similarity with a spatio-temporal category. The XAI-SHAP method instead provides justification to prioritise gauging and monitoring efforts in those unmatched catchments to better understand the spatial temporal dynamics of DIN in coastal areas that those unmatched catchments were located in. For the ungauged catchments that did match to gauged ones using the XAI-SHAP method, the subsequent ANN-WQ simulator development and case study to test the second hypothesis, found prior discrimination of data included in the training dataset, based on the spatio-temporal category of the ungauged catchment, improved performance of the ANN-WQ simulator in all scenarios tested. It was, however, an unexpected finding that, after the spatio-temporal discrimination by category was first applied, inclusion of Original Vegetation, Ecounit or Land Use variables had insignificant influence on results. Findings that emerged throughout this study therefore built nuance to our expected hypothesis 3 whereby although a trained ANN-WQ simulator successfully simulated DIN in the unsupervised scenario, it was the knowledge provided by original vegetation data to pre-process the training datasets into categories that mattered. Implications of these findings are that XAI-SHAP coupled with Original Vegetation data has demonstrated merit for customising catchment matching to the portion of water quality datasets most likely to share similar DIN to flow regimes between gauged and ungauged catchments.

## Methods

### Study area

**Figure 4:**
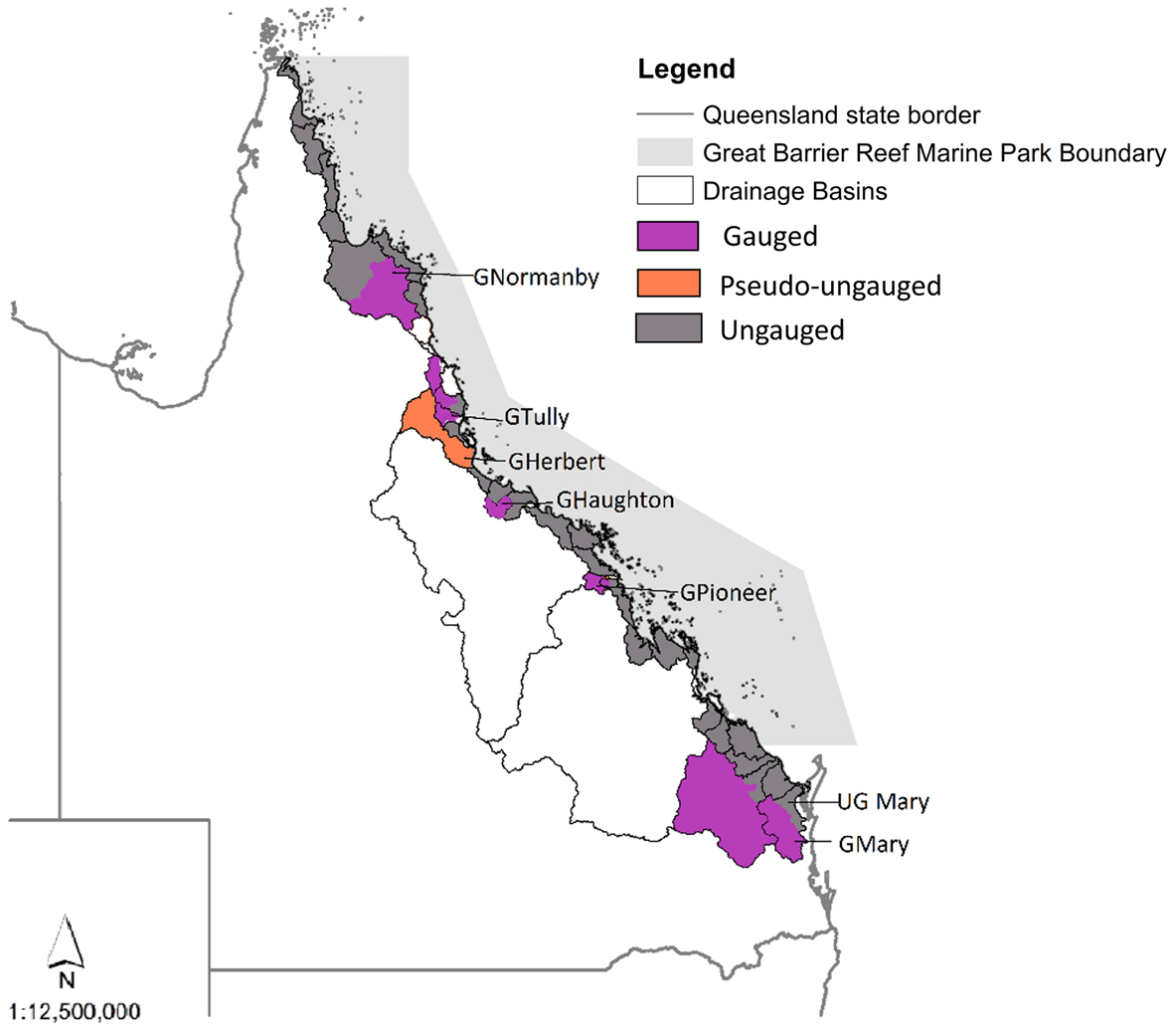
Study Area. G preceding catchment name infers a true gauged catchment. UG preceding catchment name infers ungauged catchment. Maps created by author using ArcMap 10.8.1, ungauged catchments^10^ supplied, Drainage Basins^37^ licenced under a Creative Commons—Attribution 3.0 Australia licence (CC BY 3.0 AU). © State of Queensland (Department of Environment and Science) 2023.

This study includes all catchments that flow to the Great Barrier Reef, in north-eastern Australia. Each of those catchments is referred to herein as gauged, ungauged and pseudo-ungauged as shown in Fig. 4. Respective gauging allocation, sampling frequency for DIN, and flow data availability for each of the catchments are provided in Supplementary Material Table ST3.

### Study concept

**Figure 5:**
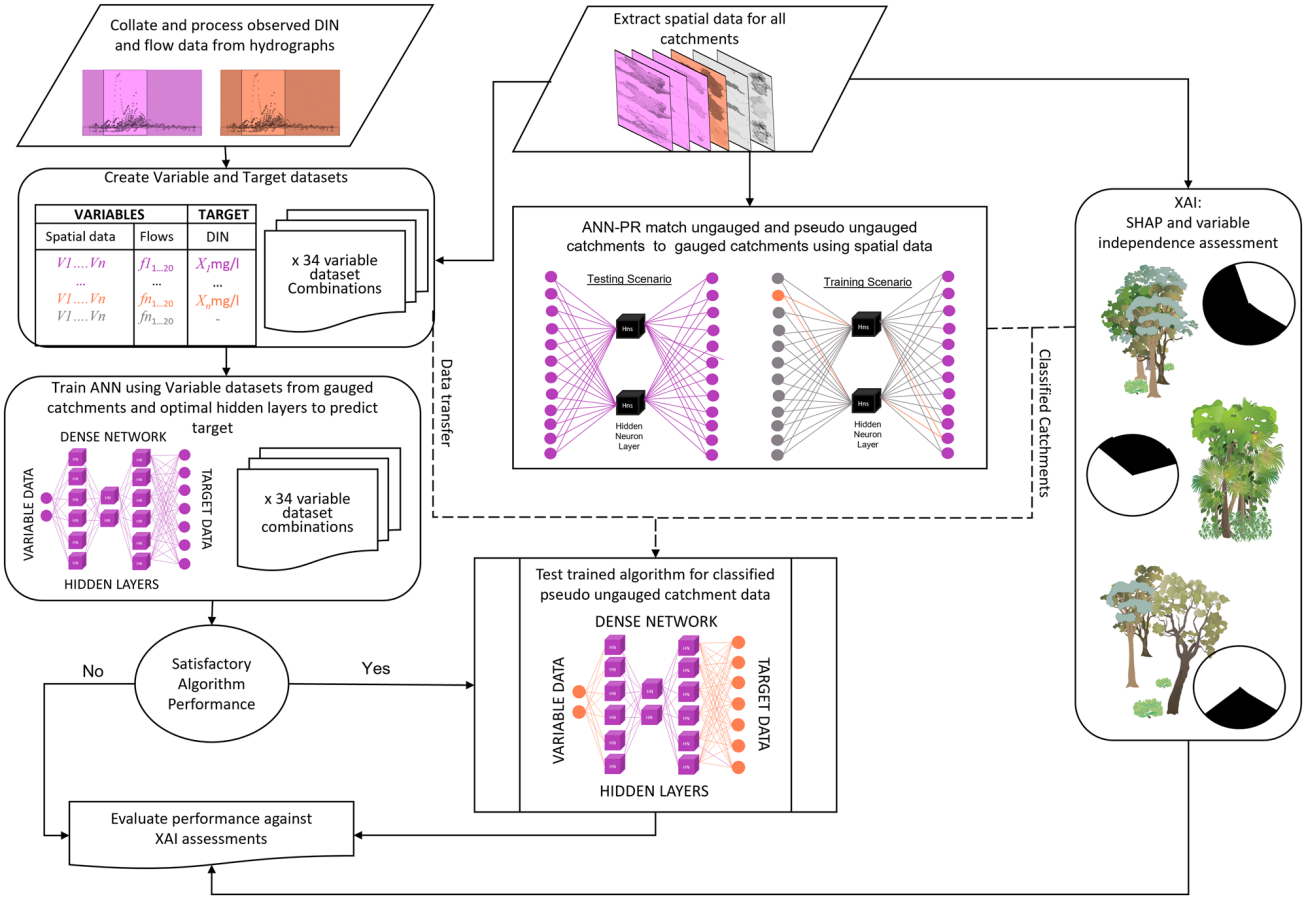
Conceptual framework of research. This framework shows data preparation for classification, process for simulating DIN for pseudo ungauged catchments, and relationship to XAI evaluation of results. Data relating to gauged catchments is represented in purple, orange represents pseudo-ungauged catchments, and ungauged catchments are represented by grey. Dashed lines show source and destination of data transfer for pseudo-ungauged catchment in the research. Vegetation images adapted from the Integration and Application Network licenced under Attribution-ShareAlike 4.0 International (CC BY-SA 4.0).

The objective of this study is to establish whether patterns in the flow and spatial variable datasets contain sufficient information to simulate Dissolved Inorganic Nitrogen (DIN), and whether forecasting capabilities can extend to new catchments, referred to in this study as pseudo-ungauged. Because the influence of every variable input and their interrelationships to overall DIN response are unknown a priori, a dense fully connected Artificial Neural Network (ANN) algorithm was developed to trial the proof of concept approach. Algorithms were trained for a number of dataset arrangements and their performance metrics were compared to quantify the viability of the novel forecasting/data transfer concept within the Artificial Intelligence modelling environment. A workflow conceptualising the research approach is shown in Fig. 5 below.

### Study dataset

Observed water quality data and flow records for those gauged and pseudo-ungauged catchments are from locations listed in Supplementary Material Table ST3. This data was sourced from Queensland State Government and was cleaned, transformed and flows arranged as detailed in our foundational research^26^. The spatial extent of gauged areas for catchments evaluated in this study are consistent with Khan et al.^13^. DIN records were collected at irregular frequencies depending on flows for each gauged catchment as detailed in Supplementary Material Table ST3. To overcome a large number of NaN values within a time series arrangement for the dataset, daily average stream and baseflows for 90 days preceding each DIN record were allocated as 90 separate column variables each on the same dataset row position as the corresponding DIN record as 1 day prior, 2 day prior….90 day prior. 90 days prior flows capture a full temperate climate season preceding each DIN record and were shown by cross correlations to be sufficient to capture residual information^60^. The water quality and flow datasets were duplicated then partitioned as outlined in O’Sullivan et al.^30^ for wet season/increasing flows, dry season/retreating flows, and all flows/seasons to capture spatio-temporal influences.

Spatial data for all gauged and ungauged portions of catchments in the study area, were extracted from Queensland Government Q-Spatial mapping platform, as per the methods described in O'Sullivan et. al.^26^. The three separate spatial datasets were created as the proxy drivers of DIN. These included: Land use to represent human biotic influence which included 6 variables^61^, Original Vegetation consisting of 38 variables^31^ intended as a parsimonious biotic response proxy for natural DIN responses across the catchment^26,62^, and Ecounit which was a created via a combination of Land use and Original Vegetation and resulted in 179 variables. The area of variables for each catchment was established via clipping the spatial datasets to the catchment boundaries and extracting corresponding data tables from ArcGIS. The area of the gauged catchments extended only to the gauged monitoring point, the ungauged portion was created as a sub-catchment polygon for all areas that drain to the catchment’s waterway downstream of the gauged monitoring point, or for fully ungauged catchments. For each catchment, the spatial dataset was duplicated to match the number of data rows to the same number of DIN records in each catchment dataset. For the pseudo-ungauged datasets, the number of spatial dataset rows were duplicated to match to the number of daily average flow records available.

A master dataset was created by joining the preceding flow and spatial dataset to create the training variables dataset, and the corresponding DIN data allocated as the target dataset. All data in each dataset was then normalised. Scenario datasets were then created by extracting subsets of data from the master dataset as detailed in Table 2.

**Table 2:** Summary of data included in scenario datasets.

Dataset type	Purpose	Catchments included	Dataset reference	Variables (discriminated by:)				Target data	
				LU spatial variables	OV spatial variables	EU spatial variables	90 day prior flows	Corresponding DIN	Corresponding catchment
1. Spatial Classification Training Datasets	Training algorithms to match spatial data to corresponding gauged catchment	All gauged	1. GCAFALU	✓	–	–	–	–	✓
			1.GCAFAOV	–	✓	–	–	–	✓
			1.GCAFAEU	–	–	✓	–	–	✓
			1.GCAFAEULUOV	✓	✓	✓	–	–	✓
2. Spatial Classification Testing Datasets	Classify pseudo-ungauged and ungauged Catchments to gauged catchment	Individual ungauged/pseudo-ungauged	2.CAFALU	✓	–	–	–	–	Hidden
			2.CAFAOV	–	✓	–	–	–	Hidden
			2.CAFAEU	–	–	✓	–	–	Hidden
			2.CAFAEULUOV	✓	✓	✓	–	–	Hidden
3. ANN-WQ simulator development- training datasets	Establish whether recognisable patterns exist in the datasets to forecast DIN	All gauged together (non-discriminated)	3.GCAFALU	✓	–	–	✓	✓	–
			3.GCAFAOV	–	✓	–	✓	✓	–
			3.GCAFAEU	–	–	✓	✓	✓	–
			3.GCAFAEULUOV	✓	✓	✓	✓	✓	–
		All gauged—individual (Discriminated by catchment)	3.G$\text {C}_{{\text{i}_1}...{\text{i}_{\text n}}}$FALU	✓	–	–	✓	✓	–
			3.G$\text {C}_{{\text{i}_1}...{\text{i}_{\text n}}}$ FAOV	–	✓	–	✓	✓	–
			3.G$\text {C}_{{\text{i}_1}...{\text{i}_{\text n}}}$ FAEU	–	–	✓	✓	✓	–
			3.G$\text {C}_{{\text{i}_1}...{\text{i}_{\text n}}}$ FAEULUOV	✓	✓	✓	✓	✓	–
		Gauged Catchments grouped by Spatio-Temporal Category (Discriminated by Category 1, Category 2 or Category 3)	3.GC_1…3_F_1…3_LU	✓	–	–	✓	✓	–
			3.GC_1…3_F_1…3_OV	–	✓	–	✓	✓	–
			3.GC_1…3_F_1…3_EU	–	–	✓	✓	✓	–
			3.GC_1…3_F_1…3_EULUOV	✓	✓	✓	✓	✓	–
4. ANN-WQ Simulator-Trial Datasets	Evaluate suitability of matched dataset for data transfer to pseudo-ungauged catchment for DIN simulation purposes	Individual gauged and pseudo-ungauged catchment	4.$\text {C}_{{\text{i}_1}...{\text{i}_{\text n}}}$FALU	✓	–	–	✓	Hidden	–
			4.$\text {C}_{{\text{i}_1}...{\text{i}_{\text n}}}$ FAOV	–	✓	–	✓	Hidden	–
			4.$\text {C}_{{\text{i}_1}...{\text{i}_{\text n}}}$ FAEU	–	–	✓	✓	Hidden	–
			4.$\text {C}_{{\text{i}_1}...{\text{i}_{\text n}}}$ FAEULUOV	✓	✓	✓	✓	Hidden	–
		Gauged and pseudo-ungauged catchments grouped by categories	4.C_1…3_F_1…3_LU	–	✓	–	✓	Hidden	–
			4.C_1…3_F_1…3_OV	–	✓	–	✓	Hidden	–
			4.C_1…3_F_1…3_EU	–	✓	–	✓	Hidden	–
			4.GC_1…3_F_1…3_EULUOV	✓	✓	✓	✓	Hidden	

Variations in each dataset intended to evaluate the influence of spatial data or flow variable toward DIN response.

*G* = Gauged, *C* Catchment, *F* Flows, *A* All, *LU* LandUse, *OV* Original Vegetation, *EU* Ecounits $C_{i_1...i_n}$ reference to individual catchments.

### Classifying gauged catchments to ungauged and pseudo-ungauged catchments

The novel aspect of this research is establishing whether pseudo-ungauged, and ungauged catchments share spatial data similarities suitable for classifying to gauged catchment classifiers, and for water quality classification data transfer purposes. Our previous studies used ANN-PR to classify only the gauged catchments together using the same spatial variables used in this study^26,30^. XAI evaluations of those datasets provide explainability to the corroborating ANN-PR results for both spatial data and water quality classification^30^. Here, we explore, for the first time, extending that classification approach beyond the gauged portion of the study area to classify catchments of the ungauged and pseudo-ungauged areas to gauged catchments. The method is therefore extending the ANN-PR approaches of our previous studies to now evaluate which gauged catchments the pseudo-ungauged and ungauged catchments classify to, and evaluate whether XAI explainability applies to the ANN-PR matches. To accomplish this, we apply a combination of the ANN-PR approach used in our previous studies and XAI explainability coupled with SHAP^36^ (XAI-SHAP) to evaluate the similarities between the catchments. This allows classification of catchments that have not been gauged, based on the similarities between the gauged catchments, and provide a better understanding of the underlying similarities between the catchments. Importantly, inclusion of the XAI-SHAP method demonstrates whether the sufficient underlying similarity is likely to exist between the proxy drivers of DIN in the gauged and ungauged catchments for the purpose of data transfer.

### Spatial classification using ANN-PR

This step used a similar approach explained in detail in our previous studies, however this time we trained the ANN-PR tool on all 11 gauged catchments, and introduced the spatial variable data for the ungauged catchments in the unsupervised environment, to force a match to one of the 11 gauged catchments. A 100-fold duplicate of each spatial variable in each gauged catchment was used to estimate the percentage match between the ungauged catchments and the gauged catchments. We then trained the ANN-PR classification tool in a supervised environment by applying the gauged catchment classification training datasets to standard codes extracted from “*MATLAB 2020a (The MathWorks Inc., 2020) Deep Learning toolbox (*Fig. 4*). The code used is a two-layer feedforward network, with sigmoid transfer function in the hidden layer, and softmax transfer function in the output layer (The MathWorks Inc. 2020)”*^63^*.* For the spatial datasets, heuristics and previous knowledge for the gauged data spatial dataset meant that an architecture of 3 hidden neurons were used to set the classification training architecture for this model. Data were split within the coding architecture to 70% for network training, 15% network validation and 15% network testing. In the training phase, the network is designed to match spatial data variables for each row in the dataset to one of the 11 the gauged catchment categories the spatial data is sourced from. The network architecture is set such that training continues towards minimisation of cross entropy and stops once mean square error elevates above its minimum pivot point at which point the ANN-PR algorithm achieves optimal performance^64^. Optimal performance is for each of the 100 replicates of spatial data to allocate to the catchment category the data belonged to in the validation and testing phase.

Testing datasets were separately introduced in an unsupervised environment to the optimised classification algorithm trained to match spatial data to only one of the 11 gauged catchments. Spatial variables for each ungauged or pseudo-ungauged catchment were duplicated 100 times so that the catchment the ungauged or gauged spatial dataset was classified to was based on 100 replicates. The algorithm forces each of the 100 rows of spatial data variables the ungauged or pseudo-ungauged catchment to match to one of the 11 classifiers in the trained environment. This approach was repeated for the Land use, Original Vegetation and Ecounit spatial datasets for all 41 ungauged and pseudo-ungauged catchments. The gauged catchment with allocations of more than half the records for each gauged or ungauged catchment was deemed classified for the respective dataset.

### Identifying variable feature independence in both gauged and ungauged catchments

The purpose of XAI, is to deduce the combination of variables most likely to have resulted in the classification between two catchments. To verify that the forced matches between gauged and ungauged catchments using ANN-PR were explainable, we therefore extend the additive deviation approach from previous work^30^ to spatial variables for all catchments, shown in Eq. (1), and graphed the top 10%^30^.

1 $${\text{D}}_{{\text{s}}} = {\text{ A}}_{{\text{s}}} - {\text{A}}_{\forall }$$

where: D: deviation of spatial dataset variable. A: proportional area of variable (A = area of variable /total catchment area), S_:_ subject variable, ∀_:_ all dataset variables excluding S.

Variables in the top 10%deviated from the mean were then graphed and visually compared for similarities between the deviated variables for gauged and ungauged catchments sharing similar combinations of deviations were categorised together. Because the Original Vegetation dataset had previously been shown to explain the ANN-PR matches between the gauged catchments^30^ it was used directly in this study. Geology and landform has also been demonstrated as a fundamental driver of nitrate in hydrological processes^48^, therefore in this study we also further scrutinised influences of the original vegetation dataset by breaking each variable down into its separate landform type and floristic structure descriptor as described by the data authority^31^ to better visualise hydrological drivers of results.

### Training ANN to forecast DIN in a supervised environment

An Artificial Neural Network water quality (ANN-WQ) simulator was developed to facilitate a rapid assessment of the similarity of matched catchments for DIN. The ANN-WQ simulator was intended for rapid comparison purposes only, and therefore method optimisation was outside the catchment classification scope intended for this research. Similarity between catchments was evaluated by the comparative accuracy of DIN simulations generated for catchments depending on the dataset scenario included in the ANN-WQ simulator training phase.

For each gauged catchment dataset, a Dense Deep Learning feed forward network was created in Matlab. The dense fully connected learning approach was selected to facilitate for all data relationships to be considered, to maximise the pattern recognition ability within the dataset, timesteps of data are still captured in variables as the corresponding time-date number. This architecture was resource intensive and therefore a ReLU hidden layer activation was included due to its superior ability to deal with weights and bias over large intensity variations, as could be expected in the dataset^65,66^.

Training datasets involved a data set split of 80% Training, 10% Verification and 10% Testing. Development of the Dense Deep Learning feed forward network began with a trial and error phase to scope for functionality at the default hidden neurons (< 10). To overcome inadequate complexity and dimensionality within datasets, trials of 1 to 1000 hidden layers were then undertaken for each dataset to identify the best performing hidden layer network suited to the training dataset^67^. Trialling up to 1000 hidden layers on big data creates heavy computing demands, therefore, Adam optimiser was selected for its minimal memory usage benefits whilst also addressing sparse gradients and non-stationary objectives^68^. The model performance metrics comprised of RMSE, MSE, Nash Sutcliffe Efficiency, Peak Deviation and Correlation as R^2^ were recorded for each of the hidden layer trials, and the algorithm with the best performance metrics evaluated for the optimal hidden neuron and for pass or fail of satisfactory performance criteria. The performance metrics equations in Table 3 identify the corresponding satisfactory performance criteria for each. For this research, the ANN-WQ simulator was used to validate whether DIN patterns were detectable. Therefore, performance criteria that identified whether the results were satisfactory or not as nominated in Table 3 were selected to remain consistent with satisfactory performance criteria for water models published elsewhere^69–73^.

**Table 3:** Performance Metrics and nominated criteria for ANN_WQ simulation scenarios.

Performance metric	Equation	Satisfactory criteria
Correlation Coefficient (R^2^)	${R}^{2}{=\left(\frac{\sum_{i=1}^{N}\left({Y}_{i}^{obs}-{{Y}_{mean}}^{obs}\right)\left({Y}_{i}^{sim}-{{Y}_{mean}}^{sim}\right)}{\sqrt{\sum_{i=1}^{N}{\left({Y}_{i}^{obs}-{{Y}_{mean}}^{obs}\right)}^{2}}\sqrt{\sum_{i=1}^{N}{\left({Y}_{i}^{sim}-{{Y}_{mean}}^{sim}\right)}^{2}}}\right)}^{2}$ Equation 2^69^	> 0.5
Nash–Sutcliffe coefficient (NSE)	$NSE=1- \left\lceil\frac{\sum_{i=1}^{N}{\left({Y}_{i}^{obs}-{Y}_{i}^{sim}\right)}^{2}}{\sum_{i=1}^{N}{\left({Y}_{i}^{obs}-{Y}_{i}^{mean}\right)}^{2}}\right\rceil$ Equation 3^54,70^	> 0.5
Willmotts index (d)	$d=1- \left\lceil\frac{\sum_{i=1}^{N}{\left({Y}_{i}^{obs}-{Y}_{i}^{sim}\right)}^{2}}{\sum_{i=1}^{N}{\left(\left|{Y}_{i}^{sim}-\right.\left.{{Y}_{mean}}^{obs}\right|+\left|\left|{Y}_{i}^{obs}-\right.\left.{{Y}_{mean}}^{sim}\right|\right.\right)}^{2}}\right\rceil$ Equation 4^71^	> 0.5
Root mean square error (RMSE)	$RMSE= \sqrt{\frac{1}{N}\sum_{i=1}^{N}{\left({Y}_{i}^{sim}-{Y}_{i}^{obs}\right)}^{2}}$ Equation 5^69,72^	Lowest
Peak percentage deviation (pde)	$100\sum_{i=1}^{N}\frac{1-{Y}_{max}^{sim}}{{Y}_{max}^{obs}}$ Equation 6^73^	< ± 25
Mean absolute error	$MAE= \frac{1}{N}\sum_{i=1}^{N}{|Y}_{i}^{sim}-{Y}_{i}^{obs}|$ Equation 7^72^	Lowest

Where: *N* = number, *i* = iteration, *Y*_*i*_^*obs*^ = Observed data, *Y*_*i*_^*sim*^ = target data from model simulation, *Y*^*mean*^_*sim*_ = mean of the simulation, *Y*^*mean*^_*obs*_ = mean of observed, *Y*^*max*^_*obs*_ = maximum of observed, *Y*^*max*^_*sim*_ = maximum of simulation.

To compare model DIN forecasts against observed DIN forecasts, the algorithm was rerun with the optimal number of hidden layers for every dataset. We normalized all data for graphing so we could compare forecasting potential across the different datasets.

### DIN forecasting potential for classified pseudo-ungauged catchments

The trained algorithms that met minimum satisfactory performance criteria as well as demonstrating a simulation ability in the supervised environment were then used in an unsupervised environment to simulate DIN for their respective classified pseudo-ungauged catchment datasets based on flow inputs. For the data available, only the pseudo-ungauged Herbert was suitable for evaluation for the study and is evaluated as a case study within this article as proof of concept. For this study, scenario datasets evaluated included the ANN-WQ simulation results for the pseudo-ungauged catchment trained on the matched gauged catchment, ANN-WQ simulator trained using all gauged catchment data, and ANN-WQ simulator trained using data from the matching spatio-temporal category. Performance metrics for each scenario were then collated and visualised in a dart plot. To create the dart plot, performance metrics were adjusted using Eq. (8) to make zero the target score. This equation has not been scaled for the impact each performance metric has towards the accuracy of the model, but is developed here for rapid comparison of overall scenario performance.

8 $${\cup }_{{PC}_{k}}={\sum }_{{\in }_{{PC}_{1\dots .n}}}^{{\in }_{{PC}_{1}}}k,$$

where: PC = Performance Criteria, K = unsupervised portion of ANN-WQ simulation scenario, 1….n = a performance criteria adjusted to make zero target i.e.{R^2^_c_, NSE_c_, d_c_, RMSE, MAE,Pdv_c_}.

Where:

R^2^_c_ = 1 − R^2^.

NSE_c_ = 1 − NSE.

pde_c_ = $$\left(pde \,\text{if pde>0}|-pde \,\text{if Pdv<0}\right)\times 0.01$$

### Supplementary Information

Supplementary Information.

## Data Availability

The datasets analysed during the current study are available from the corresponding author on reasonable request, as well as in raw form from the following public sources: Observed water quality and flow records^37,74^—Queensland Government Water Monitoring Information Portal: https://water-monitoring.information.qld.gov.au/. Original Vegetation^31^—Pre-clearing broad vegetation groups—Queensland (v4): http://qldspatial.information.qld.gov.au/catalogue/custom/search.page?q=%22Pre-clearing broad vegetation groups - Queensland%22. Land Use^61^—Land use mapping—1999 to 2017—Queensland http://www.qld.gov.au/environment/land/vegetation/mapping/qlump/.
