# Author Correction: Explainable AI approach with original vegetation data classifies spatio-temporal nitrogen in flows from ungauged catchments to the Great Barrier Reef

**DOI:** 10.1038/s41598-023-48938-0

**Published:** 2023-12-18

**Authors:** Cherie M. O’Sullivan, Ravinesh C. Deo, Afshin Ghahramani

**Affiliations:** 1 University of Southern Queensland, Toowoomba, QLD 4350 Australia; 2 School of Mathematics, Physics and Computing, University of Southern Queensland, Springfield, QLD 4300 Australia; 3 Center for Applied Climate Sciences, University of Southern Queensland, Toowoomba, QLD 4350 Australia; 4 Present Address: Department of Environment and Science, Queensland Government, Rockhampton, QLD 4700 Australia

Correction to: *Scientific Reports* 10.1038/s41598-023-45259-0, published online 24 October 2023

The original version of this Article contained an error in the Results section, under the subheading ‘Verification of catchment classification for DIN similarities’, where two instances of the unit ‘mg/L’ were incorrectly stated as “m/L” and “g/L”, respectively.

“While simulated peaks were under estimated in all cases, a review of the raw data identified that the maximum nitrogen concentration in the dataset for Herbert Catchment was 1.8105 m/L, which is the highest historical record, plus two additional peaks ranging between 1.320 g/L and 1.694 mg/L.”

now reads:

“While simulated peaks were under estimated in all cases, a review of the raw data identified that the maximum nitrogen concentration in the dataset for Herbert Catchment was 1.8105 mg/L, which is the highest historical record, plus two additional peaks ranging between 1.320 mg/L and 1.694 mg/L.”

The original Article has been corrected.

