# Supplementary material for: Explainable AI approach with original vegetation data classifies spatio-temporal nitrogen in flows from ungauged catchments to the Great Barrier Reef
Source: Sci Rep. 2023 Oct 24;13:18145. doi: 10.1038/s41598-023-45259-0 (PMC10598196; doi:10.1038/s41598-023-45259-0)
Supplement: Supplementary file 1 — Supplementary Information. [file 41598_2023_45259_MOESM1_ESM.pdf]

Supplementary Material S1: Abbreviations

A=All

ANN = Artificial Neural Network

C=Catchment

Category 1=Catchments with similar DIN patterns during increasing flows and rainy season.

Category 2=Catchments with year round similar DIN patterns.

Category 3= Catchments with similar DIN patterns during retreating flows and dry season.

CM=Mary Catchment

d=Willmotts Index

DIN = Dissolved Inorganic Nitrogen

EU=Ecounits

F=Flows

F1=Category 1 flows (Wet season/increasing flows)

G=Gauged

LU=Land use

Match=Catchments paired together for their similarities

MSE=Mean Square Error

NSE=Nash Sutcliffe Efficiency

obs=observed data

OV=Original Vegetation

OW=Open Woodlands

pde=Peak Percentage Deviation

PR=Pattern Recognition

ReLU=Rectified Linear Units

R^2=regression coefficient

RMSE=Root Mean Square Error

SHAP= Shapley Additive exPlanations

sim=simulation

WQ=Water Quality

WT=Wet Tropics

XAI=eXplainable Artificial Intelligence

Supplementary Material Figure SF1:

ANN_WQ simulator development. Example simulation results during code development for DIN for individual catchments identified as a Category 1 spatio temporal catchment vs Category 2 spatio temporal catchment. Simulations flatlined for testing of Category 1 catchments trained on their own data, while simulations were possible for Category 2 catchments.

a) Category 1 spatio temporal catchment

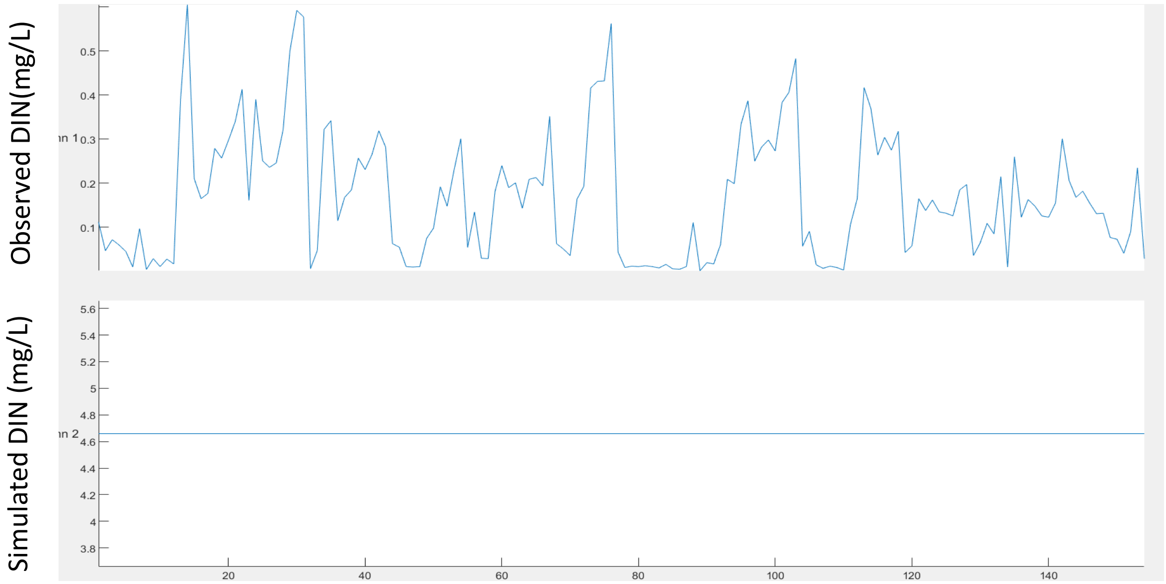

b) Category 2 spatio temporal catchment

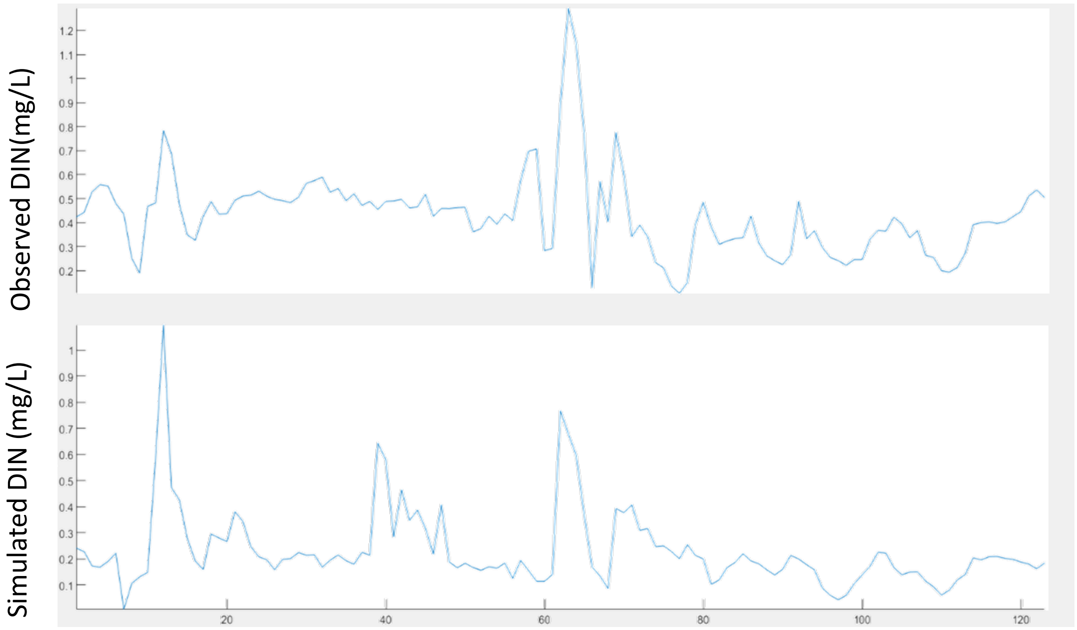

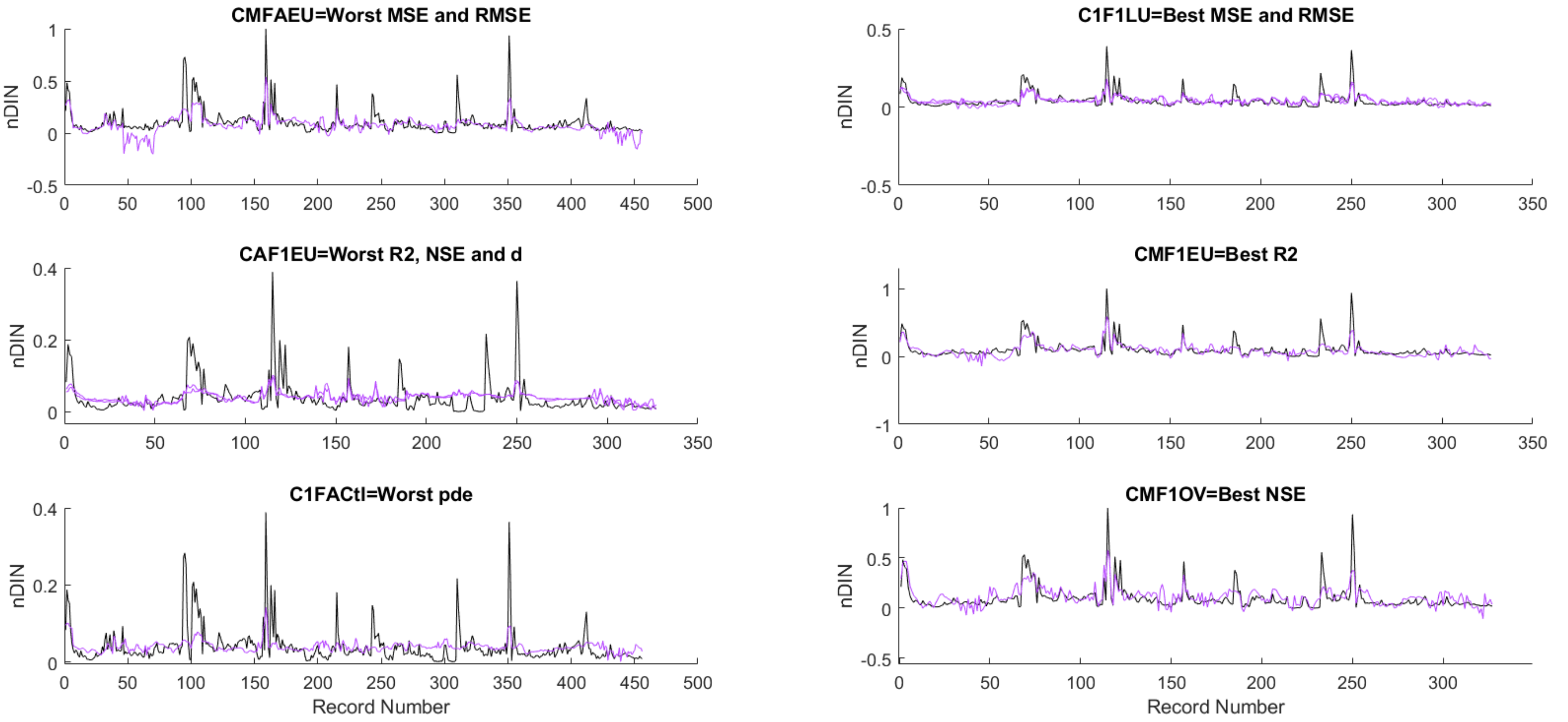

Supplementary Material Figure SF2: Visualisation of best and worst performing scenarios for DIN simulation of Herbert. Blackline represents observed (true) data, purple lines are the simulated data for scenario that matched the best/worst performance criteria, as identified by the heading for each graph. C= catchment data included in ANN_WQ simulator training scenario, F= flow data included in ANN_WQ simulator training scenario, M = Mary Catchment Data, A=All catchment data, 1 = increasing flows and wet season data, EU= Ecounit, OV= Original Vegetation, LU= Land use,. MSE = Mean Square Error, RMSE= Root Mean Square Error, R2= Correlation Coefficient, NSE= Nash-Sutcliffe coefficient, d= Willmotts Index.

Supplementary Material Table ST1: Kruskal Wallis Test for independence of performance criteria distribution outputs from the ANN_WQ simulator. Independence exists where datasets are first discriminated to only include catchments and flow data representative of the spatio temporal regime. Inclusion of spatial data have insignificant influence where included in the training dataset.

Hypothesis	X	Sig.[a,b]	Decision
Category Groups (i.e. All, Cat 1, Cat 2, Cat 3 and Herbert Trial) have same distribution of performance criteria X	MSE	0.025	***Reject the null hypothesis.***
	R^2	0.003	***Reject the null hypothesis.***
	NSE	0.003	***Reject the null hypothesis.***
	d	0.003	***Reject the null hypothesis.***
	RMSE	0.025	***Reject the null hypothesis.***
	pde	0.045	***Reject the null hypothesis.***
Spatial Data Scenarios (i.e. Control, EU, LU, OV, All) have same distribution of performance criteria X	MSE	0.725	Retain the null hypothesis.
	R^2	0.951	Retain the null hypothesis.
	NSE	0.981	Retain the null hypothesis.
	d	0.967	Retain the null hypothesis.
	RMSE	0.725	Retain the null hypothesis.
	pde	0.453	Retain the null hypothesis.

a. Significance level is 0.05

b. Asymptotic significance is displayed

Supplementary Material Table ST2: Performance metrics for simulations of DIN for the pseudo ungauged catchment (Herbert) generated from an ANN_WQ simulator developed using the differing training dataset scenarios. Scenario abbreviation: CAll= data from all catchments, CM=data from Mary only, FAll = all flow data included, F1= flows discriminated to Category 1 spatio temporal regime of Wet Season Increasing Flows, Ctl = no spatial data included, EU= Ecounit, LU= Land Use, OV= Original Vegetation spatial data included .Performance metrics and corresponding scenario styled in: ***red bold italic*** = best performing scenario; **black bold** = poorest performing scenario; grey= failed to meet minimum standard for the corresponding performance metric. Results show scenarios trained on the single catchment matched using ANN_PR for Ecounits (Mary) and flow variables discriminated to the Category 1 spatio temporal regime of Wet Season Increasing Flows collectively achieved the best performing performance for R2 (EU), NSE (OV) and Willmots d efficiency and pde (LU). With the exception of C1F1LU and C1F1OV, and CAllFAllOV, training datasets only including data from the individual matched catchment achieved satisfactory NSE scores.

Scenario	Category	Flows	Spatial	Best score from the 1000hn trial for :					
			Scenario	MSE	R2	NSE	d	RMSE	pde
C1F1Ctl	1	1	Ctl	0.00126	0.67389	0.44019	0.69366	0.03556	-1.282
C1F1EU	1	1	EU	0.00127	0.69564	0.43751	0.69752	0.03565	45.8329
C1F1LU	1	1	LU	***0.00099***	0.78699	0.5601	0.78032	***0.03153***	21.198
C1F1OV	1	1	OV	0.00108	0.77024	0.52022	0.74909	0.03292	-2.5231
**C1FAllCtl**	**1**	**All**	**Ctl**	0.00147	0.59903	0.29789	0.51612	0.03831	**58.1074**
C1FAllEU	1	All	EU	0.00128	0.65115	0.38713	0.6672	0.0358	-32.352
C1FAllLU	1	All	LU	0.00112	0.71513	0.46336	0.70666	0.0335	48.6003
C1FAllOV	1	All	OV	0.00117	0.68981	0.44003	0.70817	0.03422	49.7629
CAllF1Ctl	All	1	Ctrl	0.00155	0.62667	0.31176	0.55666	0.03943	44.3145
**CAllF1EU**	**All**	**1**	**EU**	0.00163	**0.58198**	**0.277661**	**0.51364**	0.04040	4.47167
CAllF1LU	All	1	LU	0.00149	0.6381	0.33853	0.59382	0.03866	40.5623
CAllF1OV	All	1	OV	0.00155	0.61461	0.31426	0.55621	0.03936	9.97363
CAllFAllCtl	All	All	Ctl	0.00116	0.70186	0.44447	0.70299	0.03408	40.0596
CAllFAllEU	All	All	EU	0.00109	0.72895	0.47703	0.7417	0.03307	0.83082
CAllFAllLU	All	All	LU	0.00106	0.75433	0.49383	0.73251	0.03253	30.7716
CAllFAllOV	All	All	OV	0.00102	0.74994	0.50971	0.74708	0.03201	13.9193
CMF1Ctl	Mary	1	Ctl	0.00607	0.77443	0.59629	0.84445	0.07791	-0.0109
CMF1EU	***Mary***	***1***	***EU***	0.00586	***0.79875***	0.61034	0.83047	0.07654	-0.389
CMF1LU	***Mary***	***1***	***LU***	0.00574	0.78762	0.61823	***0.84946***	0.07576	***-0.012***
CMF1OV	***Mary***	***1***	***OV***	0.00571	0.79165	***0.62034***	0.84499	0.07555	0.11908
CMFACtl	Mary	All	Ctl	0.00673	0.72241	0.51473	0.78144	0.08201	0.08782
**CMFAEU**	Mary	All	EU	**0.00679**	0.74263	0.51034	0.77184	**0.08238**	-2.1616
CMFALU	Mary	All	LU	0.00642	0.7459	0.53679	0.78972	0.08012	-10.167
CMFAOv	Mary	All	OV	0.0065	0.73849	0.53101	0.78007	0.08062	16.3765

Supplementary Table ST3: Gauging allocation, DIN, and flow data availability for each of the catchments[75,76].

Catchment flowing to Great Barrier Reef	Original Vegetation Data	Landuse Data	Gauging allocation	Gauging station ID for observed data	Gauging station Latitude (decimal °)	Gauged station Longitude (decimal °)	Catchment area (km^2)	Natural Resource Management Region	DIN (mg/L) at daily streamflows (averaged from hourly)for:			Mean DIN	Standard Deviation of DIN (mg/L)	DIN Record Period	DIN sampling frequency	Number of records in DIN record period
									Min	Max	Mode					
CurtisIsland	✓	✓	Ungauged	-	-	-	564	Fitzroy	-	-	-		-	-	-	-
Jardine	✓	✓	Ungauged	-	-	-		Cape York	-	-	-		-	-	-	-
JackyJacky	✓	✓	Ungauged	-	-	-	3,102	Cape York	-	-	-		-	-	-	-
GOlivePascoe	✓	✓	Psudo-Ungauged	102102A	-12.657785	143.050145	132	Cape York						-	-	-
UGOlivePascoe	✓	✓	Ungauged	-	-	-		Cape York	-	-	-		-	-	-	-
Gstewart	✓	✓	Psudo-Ungauged	104001A	-14.167489	143.394002	471	Cape York						-	-	-
UGStewart	✓	✓	Ungauged	-	-	-	2,342	Cape York	-	-	-		-	-	-	-
GNormanby	✓	✓	Gauged	105107A	-15.46	144.56	12,828	Cape York	0.012<br>0.011<br>0.069<br>0.017<br>0.006<br>0.016<br>0.005	0.013	148.24	0.055	0.128	3/10/2006-<br>25/08/2017	Events<br>Jan-March	244
UGNormanby	✓	✓	Ungauged	-	-	-	11,992	Cape York	-	-	-		-	-	-	-
Jeannie	✓	✓	Ungauged	-	-	-	3,711	Cape York	-	-	-		-	-	-	-
Endeavour	✓	✓	Ungauged	-	-	-	2,214	Cape York	-	-	-		-	-	-	-
Gdaintree	✓	✓	Psudo-Ungauged	1080025A	-16.1796	145.2819	911	Wet tropics						-	-	-
UGDaintree	✓	✓	Ungauged	-	-	-	1,217	Wet tropics	-	-	-		-	-	-	-
Mossman	✓	✓	Ungauged	-	-	-	475	Wet tropics	-	-	-		-	-	-	-
GBarron	✓	✓	Gauged	110001D	-17.05	145.51	1,950	Wet tropics	0.0045	0.154	0.004<br>0.235	0.117	0.091	19/01/06-<br>15/09/17	Regular<br>(1)<br>monthly,<br>Events<br>Jan-March	318
UGBarron	✓	✓	Ungauged	-	-	-	250	Wet tropics	-	-	-		-	-	-	-
GMulgraveRussell	✓	✓	Psudo-Ungauged	111007A	-17.133361	145.764556	523.19	Wet tropics	-	-	-		-	-	-	-
MulgraveRussell	✓	✓	Ungauged	-	-	-		Wet tropics	-	-	-		-	-	-	-
G North Johnstone	✓	✓	Gauged	112004A	-17.5	145.69	926	Wet tropics	0.0035	0.157		0.147	0.073	30/01/2006-<br>15/09/2017	Infrequent<br>half<br>yearly,<br>Events<br>Jan-March	94
GSouth Johnstine	✓	✓	Gauged	112101B	-17.66	145.77	399	Wet tropics	0.009	0.044	0.103<br>0.050	0.126	0.065		Regular<br>(1)<br>monthly,<br>Events<br>Jan-March	414
UGJohnstone	✓	✓	Ungauged	-	-	-	875	Wet tropics	-	-	-		-	-	-	-
GTully	✓	✓	Gauged	113006A	-17.87	145.72	1,386	Wet tropics	0.008<br>0.090	0.062	0.270<br>0.225	0.237	0.154	13/01/2006-<br>19/04/2018	Frequent<br>(>1)<br>monthly,	723

Catchment flowing to Great Barrier Reef	Original Vegetation Data	Landuse Data	Gauging allocation	Gauging station ID for observed data	Gauging station Latitude (decimal °)	Gauged station Longitude (decimal °)	Catchment area (km²)	Natural Resource Management Region	DIN (mg/L) at daily streamflows (averaged from hourly)for:			Mean DIN	Standard Deviation of DIN (mg/L)	DIN Record Period	DIN sampling frequency	Number of records in DIN record period
									Min	Max	Mode					
															Events Jan-March	
UGTully	✓	✓	Ungauged	-	-	-	298	Wet tropics	-	-	-		-	-	-	-
Murray	✓	✓	Ungauged	-	-	-	1,107	Wet tropics	-	-	-		-	-	-	-
Gherbert	✓	✓	Psudo-Ungauged	116006B	-18.488994	145.936037	7,490	Wet tropics		0.084		0.186002571	0.213	-	-	-
UGHerbert	✓	✓	Ungauged	-	-	-	2,348	Wet tropics	-	-	-		-	-	-	-
Black	✓	✓	Ungauged	-	-	-	1,053	NQ Dry Tropics	-	-	-		-	-	-	-
Ross	✓	✓	Ungauged	-	-	-	1,696	NQ Dry Tropics	-	-	-		-	-	-	-
GHaughton	✓	✓	Gauged	119003A	-19.72	146.81	1,807	Burdekin	0.008	0.252	0.008	0.066	0.088	20/12/2012-25/09/2017	Regular monthly, Events Jan-March	80
UGHaughton	✓	✓	Ungauged	-	-	-	2,211	Burdekin	-	-	-		-	-	-	-
UGBurdekin	✓	✓	Ungauged	-	-	-	128445**	Burdekin	-	-	-		-	-	-	-
Don	✓	✓	Ungauged	-	-	-	3,698	Mackay Whitsunday	-	-	-		-	-	-	-
Proserpine	✓	✓	Ungauged	-	-	-	2,466	Mackay Whitsunday	-	-	-		-	-	-	-
GOConnell	✓	✓	Gauged	124001B	-20.77	0.008<br>0.090	336	Mackay Whitsunday	0.008<br>0.090	0.062	0.008<br>0.090	0.109	0.14	25/01/2007-24/08/2017	Irregularly One off Events Jan-March	87
UGOConnell	✓	✓	Ungauged	-	-	-	2,021	Mackay Whitsunday	-	-	-		-	-	-	-
GPioneer	✓	✓	Gauged	125013A	-21.23	148.74	1,464	Mackay Whitsunday	0.021<br>0.071<br>0.823<br>0.914<br>0.624<br>0.012<br>0.011	0.061	0.021<br>0.071<br>0.823<br>0.914<br>0.624<br>0.012<br>0.011	0.231	0.275	18/10/2006-13/09/2017	Frequent (>1) monthly, Events Jan-March	402
UGPioneer	✓	✓	Ungauged	-	-	-	87	Mackay Whitsunday	-	-	-		-	-	-	-
GPlane	✓	✓	Gauged	126001A	-21.24	148.94	327	Mackay Whitsunday	0.009<br>0.013	0.046	0.961<br>1.287<br>1.265	0.424	0.533	4/09/2009-26/08/2017	Regular (1) monthly, Events Jan-March	302
UGPlane	✓	✓	Ungauged	-	-	-	2,173	Mackay Whitsunday	-	-	-		-	-	-	-
Styx	✓	✓	Ungauged	-	-	-	2,959	Fitzroy	-	-	-		-	-	-	-
Shoalwater	✓	✓	Ungauged	-	-	-	3,535	Fitzroy	-	-	-		-	-	-	-
Waterpark	✓	✓	Ungauged	-	-	-	1,797	Fitzroy	-	-	-		-	-	-	-
UGFitzroy	✓	✓	Ungauged	-	-	-	139544*	Fitzroy	-	-	-		-	-	-	-
Calliope	✓	✓	Ungauged	-	-	-	2,193	Fitzroy	-	-	-		-	-	-	-
Boyne	✓	✓	Ungauged	-	-	-	2,441	Fitzroy	-	-	-		-	-	-	-
Baffle	✓	✓	Ungauged	-	-	-	3,992	Burnett Mary	-	-	-		-	-	-	-

Catchment flowing to Great Barrier Reef	Original Vegetation Data	Landuse Data	Gauging allocation	Gauging station ID for observed data	Gauging station Latitude (decimal °)	Gauged station Longitude (decimal °)	Catchment area (km^2)	Natural Resource Management Region	DIN (mg/L) at daily streamflows (averaged from hourly)for:			Mean DIN	Standard Deviation of DIN (mg/L)	DIN Record Period	DIN sampling frequency	Number of records in DIN record period
									Min	Max	Mode					
Kolan	✓	✓	Ungauged	-	-	-	2,838	Burnett Mary	-	-	-		-	-	-	-
GBurnett	✓	✓	Gauged	136007A	-25.73	151.28	30,724	Burnett Mary	0.004	0.281	0.004<br>0.119	0.161	0.318	23/10/20006-15/09/2017	Frequent (>1) monthly, Events Jan-March	400
UGBurnett	✓	✓	Ungauged	-	-	-	1,675	Burnett Mary	-	-	-		-	-	-	-
Burrum	✓	✓	Ungauged	-	-	-	3,293	Burnett Mary	-	-	-		-	-	-	-
GMary	✓	✓	Gauged	138014A	-26.19	152.49	6,863	Burnett Mary	0.017<br>0.061	0.236	0.017<br>0.061	0.201	0.204	25/09/2013-29/06/2018	Frequent (>1) monthly, Events Jan-March	176
UGMary	✓	✓	Ungauged	-	-	-	2,372	Burnett Mary	-	-	-		-	-	-	-

Citation:

75. State of Queensland Department of Environment and Resource Management (2012) State Surface water Ambient Water Quality Network WMP014 version 2.
76. State of Queensland Department of Natural Resources, Mines and Energy (2018) Surface Water Ambient Network (Water Quality) 2018-19, WMP014 version 6, June 2018.
